# Supplementary material for: COZOID: contact zone identifier for visual analysis of protein-protein interactions
Source: BMC Bioinformatics. 2018 Apr 6;19:125. doi: 10.1186/s12859-018-2113-6 (PMC5889581; doi:10.1186/s12859-018-2113-6)
Supplement: Supplementary file 2 — Software build. Executable binary file of the software tool. (ZIP 118,784 kb) [file 12859_2018_2113_MOESM2_ESM.zip › cozoid/platform/docs/testng-6.5.1-javadoc.zip!/org/testng/IMethodSelector.html]

IMethodSelector (TestNG)


---


|  |  |  |
| --- | --- | --- |
| **Overview** \| **Package** \| **Class** \| **Tree** \| **Deprecated** \| **Index** \| **Help** | |  |
| **PREV CLASS**   **NEXT CLASS** | **FRAMES**    **NO FRAMES**     **All Classes** |  |
| SUMMARY: NESTED \| FIELD \| CONSTR \| METHOD | DETAIL: FIELD \| CONSTR \| METHOD |  |


---


## org.testng Interface IMethodSelector

**All Superinterfaces:**: java.io.Serializable

---

``` public interface IMethodSelector extends java.io.Serializable ```

This interface is used to augment or replace TestNG's algorithm to
decide whether a test method should be included in a test run.
Created on Sep 26, 2005

**Author:**
:   cbeust

---

| **Method Summary** | |
| --- | --- |
| `boolean` | `includeMethod(IMethodSelectorContext context, ITestNGMethod method, boolean isTestMethod)` |
| `void` | `setTestMethods(java.util.List<ITestNGMethod> testMethods)`             Invoked when all the test methods are known so that the method selector can perform additional work, such as adding the transitive closure of all the groups being included and depended upon. |

**Method Detail**

### includeMethod

```
boolean includeMethod(IMethodSelectorContext context,
                      ITestNGMethod method,
                      boolean isTestMethod)
```

:   **Parameters:**: `context` - The selector context. The implementation of this method can invoke setHalted(true) to indicate that no other Method Selector should be invoked by TestNG after this one. Additionally, this implementation can manipulate the Map object returned by getUserData().: `method` - The test method: `isTestMethod` - true if this is a @Test method, false if it's a configuration method **Returns:**: true if this method should be included in the test run, false otherwise

---


### setTestMethods

```
void setTestMethods(java.util.List<ITestNGMethod> testMethods)
```

:   Invoked when all the test methods are known so that the method selector
    can perform additional work, such as adding the transitive closure of
    all the groups being included and depended upon.


---


|  |  |  |
| --- | --- | --- |
| **Overview** \| **Package** \| **Class** \| **Tree** \| **Deprecated** \| **Index** \| **Help** | |  |
| **PREV CLASS**   **NEXT CLASS** | **FRAMES**    **NO FRAMES**     **All Classes** |  |
| SUMMARY: NESTED \| FIELD \| CONSTR \| METHOD | DETAIL: FIELD \| CONSTR \| METHOD |  |


---
